# Scottish Medical Imaging Service - Technical and Governance controls.

**DOI:** 10.23889/ijpds.v7i3.1869

**Published:** 2022-08-25

**Authors:** Jackie Caldwell, Robert Wallace, Carole Morris, Simon Fleming, Rob Baxter, Ruairidh MacLeod, William Kerr, Donald Scobbie, Simon Rogers, Felix Ritchie, Esma Mansouri-Benssassi, Susan Krueger, Emily Jefferson

**Affiliations:** 1 Public Health Scotland; 2 University of Edinburgh; 3 NHS; 4 University of the West of England; 5 University of Dundee

## Objectives

The Scottish Medical Imaging (SMI) service provides linkable, population based, “research-ready” real-world medical images for researchers to develop or validate AI algorithms within the Scottish National Safe Haven. The PICTURES research programme is developing novel methods to enhance the SMI service offering through research in cybersecurity and software/data/infrastructure engineering.

## Approach

Additional technical and governance controls were required to enable safe access to medical images.

The researcher is isolated from the rest of the trusted research environment (TRE) using a Project Private Zone (PPZ). This enables researchers to build and install their own software stack, and protects the TRE from malicious code.

Guidelines are under development for researchers on the safe development of algorithms and the expected relationship between the size of the model and the training dataset. There is associated work on the statistical disclosure control of models to enable safe release of trained models from the TRE.

## Results

A policy enabling the use of “Non-standard software” based on prior research, domain knowledge and experience gained from two contrasting research studies was developed. Additional clauses have been added to the legal control – the eDRIS User Agreement – signed by each researcher and their Head of Department. Penalties for attempting to import or use malware, remove data within models or any attempt to deceive or circumvent such controls are severe, and apply to both the individual and their institution. The process of building and deploying a PPZ has been developed allowing researchers to install their own software.

No attempt has yet been made to add additional ethical controls; however, a future service development could be validating the performance of researchers’ algorithms on our training dataset.

## Conclusion and Relevance

The availability to conduct research using images poses new challenges and risks for those commissioning and operating TREs. The Private Project Zone and our associated governance controls are a huge step towards supporting the needs of researchers in the 21st century.

